# Attitudes and Perceptions on Advance Care Planning Among Chinese-Speaking Older Australians

**DOI:** 10.1177/10499091231200366

**Published:** 2023-09-01

**Authors:** Ling H. Yeoh, Benjamin Tan, Joel Rhee, Craig Sinclair

**Affiliations:** 1 Menzies School of Health Research, 10095 Charles Darwin University, Darwin, NT, Australia; 2 School of Nursing, Faculty of Health, 10095 Charles Darwin University, Darwin, NT, Australia; 3 School of Population Health, 7800 University of New South Wales, Sydney, NSW, Australia; 4 School of Psychology, 7800 University of New South Wales, Sydney, NSW, Australia; 5 Neuroscience Research Australia, Sydney, NSW, Australia; 6 UNSW Ageing Futures Institute, Sydney, NSW, Australia

**Keywords:** advance care planning, autonomy, Chinese-speaking, collective decision-making, culturally and linguistically diverse, end-of-life decision-making, older Australians

## Abstract

**Background:**

Current literature indicates low uptake of advance care planning (ACP) among the Chinese-speaking community in Australia. To increase the uptake of ACP among the Chinese-speaking community, a better understanding of their attitudes and perceptions on end-of-life (EOL) matters, and ACP is required.

**Objective:**

This study aimed to identify significant events and social and cultural factors that influence participants’ values and characterize the attitudes and perceptions towards ACP among older Chinese-speaking Australians.

**Methods:**

A qualitative study explored participants’ experiences through semi-structured one-to-one interviews. The interviews were conducted in Mandarin, Cantonese or English, then translated and transcribed into English. The transcripts were coded and analysed thematically.

**Results:**

Twenty participants were recruited (14 female, six male). Participants typically reported a preference to make health-related decisions autonomously. Their perspectives were grounded in past experiences of illnesses and EOL decision-making of loved ones, personal values, and perceived needs. Family dynamics and intimacy of relationships appeared to influence the role and responsibility of family members in EOL decision-making and ACP. Most participants perceived the need to engage in ACP only when encountering significant health changes or higher care needs.

**Conclusion:**

Healthcare professionals should initiate ACP discussion using culturally appropriate communication with consideration of personal values, past experiences and family dynamics. Efforts should be invested in raising public awareness of ACP within the Chinese-Australian community.

## Introduction

Advance care planning (ACP) is an ongoing process that supports adults of any age or stage of health in outlining one’s care preferences and end-of-life (EOL) wishes for a time when they no longer have the decision-making capacity or ability to communicate for themselves.^1^ ACP promotes autonomy and a person-centred approach to care decisions to ensure that people receive medical care consistent with their values, goals, and preferences.^1^ ACP may also include entrusting someone else to make a medical decision when they can no longer decide.^1^ When documented and adhered to, ACP has been reported to reduce unwanted hospitalization and aggressive treatments, reduce stress and anxiety among the staff, the patients and the family members, and avoidance of decision conflicts among family members.^2-4^

In Australia, the legal status of ACP is supported by statutory law and common law. The legislation varies between the states and territories.^5^ In New South Wales (NSW) where this study was conducted, the ACP process is supported under the common law. An ACP discussion may result in an advance care directive (ACD) which could take two forms, firstly, a legally-binding documentation of the person's preferences regarding their future care choices; and secondly, the appointment of a substitute decision-maker who could provide consent for healthcare and medical treatments on behalf of the person when their decision-making capacity is lost.^6^

Recent studies have found that only one in four older Australians aged 65 years or above has an ACD in place; and the awareness of ACP amongst community-dwelling older Australians and their families were reportedly low.^7,8^ Sinclair et al.^9^ found that among older adults aged 65 years or above, the rates of ACD completion were lower among those who were born outside of Australia than those who were born in Australia. The uptake of ACP was somewhat lower among those born in Asia compared to other birth regions such as Europe.

As Indicated by current literature, the awareness and uptake of ACP are comparatively low among people from Culturally and Linguistically Diverse (CALD) communities in Australia.^9-13^ Many older adults from CALD backgrounds encounter challenges in communicating their EOL preferences due to language barriers, lack of awareness of ACP, low health literacy, lack of social or familial support or lack of communication due to death being a taboo topic.^7,14,15^ People with life-limiting illnesses from CALD background and their carers often report unmet needs at the end of life, including information, instrumental, and emotional support needs.^10,11,15,16^ Advance Care Planning Australia is a national program funded by the Australian Government Department of Health and Aged Care to provide education and advocacy on ACP to the general public, as well as healthcare professionals.^17^ Acting as the “go to” national resources on ACP, its website content is also available in multiple languages. There has not yet been a nation-wide education or awareness program targeting the CALD communities in Australia.

In Sydney, Australia, more than one-fifth (21 per cent) of Australians speak a language other than English at home.^18^ Mandarin is the most common language, with Cantonese being the next most common. Many Australians originating from China and other Asian countries or regions self-identify as Chinese ancestry and Chinese-speaking.^18^ Death is considered a taboo topic by many migrants from Asia.^19^ Many migrants who self-identify as Chinese still uphold the philosophy of Confucianism, particularly the older generation.^20^ According to Chinese Bioethics experts, the traditional philosophy of Confucian advises against revealing the prognosis to the patients with the reason that the imminent truth would do more harm than benefit to the patient.^21^ Therefore, families, in particular, the eldest son or sibling is entrusted with the responsibility and authority to make EOL decisions in the patient's best interest.^22^ Avoidance of EOL conversation and obligation of filial piety (family take over decision making) has been identified as significant barriers for people from Asian cultures in taking up ACP and accessing palliative care.^10,11,19^ There has been no research reporting on the association between the social and cultural conditions and the perceptions and readiness for the uptake of ACP among Chinese -speaking older Australians. This research aimed to characterize attitudes towards ACP and EOL decision-making among Chinese-speaking older Australians living in Sydney.

## Methods

This study is a qualitative study with an inductive approach.^23^ Data were collected by conducting one-to-one semi-structured interviews in the participants’ preferred language – Mandarin, Cantonese or English, and then analyzed to draw thematic insights.^23,24^

We aimed to recruit 20 participants or until data saturation (i.e., no more new themes emerge). While recognizing that there will always be new information, data saturation is determined to be reached when the ability to obtain additional new information from further interviews is limited.^25^

### Culturally appropriate communication strategy

The interview guide was designed to derive meaning from the participants’ life events, social and cultural conditions, and perceptions. A de-personalized communication strategy of using another person’s story was adopted to broach the' taboo' discussion about EOL matters.^26^ Participants were told a story of how a Chinese family navigated through a common EOL scenario, followed by questions to prompt reflection on their views (see Appendix 1). The research team developed the interview guide used in this study based on the team’s experience working with Chinese-speaking patients in health and aged care settings.

The recruitment and interview processes were conducted by the first author, a bi-lingual researcher who self-identifies as Chinese and is proficient in both Mandarin and Cantonese to minimize language barrier. All communication materials, such as the participant information sheet and the consent form, were presented in traditional Chinese, simplified Chinese and English. A local service provider specializing in working with Chinese-speaking clients was consulted to ensure the translations of the materials were contextually and culturally appropriate.

### Participants and setting

Participants consisted of Chinese-speaking older Australians 65 years old and older and informal carers or family members 18 years and older living in the Sydney metropolitan area. Participants were recruited purposively through referrals made by the community and aged care providers, self-referral and snowball referrals.^27^

### Data collection

Interviews were conducted and recorded either face-to-face or via phone call. Interviews in Mandarin or Cantonese were transcribed verbatim and then translated into English by the first author who is a bilingual researcher. The final English transcripts were summarized and back-translated into the participant’s preferred language by the same author, enabling participants to validate the authenticity of the transcript summaries.^28^

### Data analysis - coding and theme formation

This study drew insights from participants’ sharing using a thematic analysis approach.^24^ During coding, we focused on the meanings or concepts behind the words or phrases instead of the word or phrase itself.^29^ Initial coding focused on breaking down the data into discrete units of meaning, then examining and comparing them. The authors reviewed and discussed discrepancies or disagreements and agreed on a preliminary coding framework, which was then used to code the remaining transcripts. Coded data were read and re-read to identify themes.^23,24,29^

### Ethics approval

The Human Research Ethics Committee of the Northern Territory Department of Health, Menzies School of Health Research approved the protocol.

## Results

Our recruitment effort stopped when we had successfully recruited 20 participants and the data had reached saturation. Twenty participants were recruited and completed the interviews in the period of May to October 2022. Four interviews were conducted face-to-face, while the others (16) were conducted via phone calls. The average length of the interviews was 45 min. Table 1 describes the participants' demographic characteristics, place of origin, and preferred language, distinguished by their roles as either older participants or carers.

**Table 1.** Demographic Characteristics of the Participants (Older Adults: Carers).

Participant characteristics	Older adults	Carers and family members
Gender (female: Male)	10:5	4:1
Mean (SD) age (years)	74 (5.3)	54 (6.9)
Mean (SD) years since migration	35 (15.2)	25 (7.7)
Living arrangement		
Living alone	7	1
Living with family/spouse	8	4
Marital status		
Divorced	2	0
Widowed	5	0
Single	0	1
Married/partnered	8	4
Place of origin		
Mainland China	5	2
Hong Kong SAR	6	2
Taiwan	2	1
Malaysia	2	0
Preferred language		
Mandarin	4	1
Cantonese	8	0
English	3	4

Recruited participants were mostly healthy community dwelling older Australians who described their functional capacity as independenct and effective in self-care. The types of illnesses or impairments reported by the participants ranged from hypertension, type 2 diabetes, chronic vertigo, osteoarthritis, Parkinson’s disease and vision impairment. None of the participants was in a critical or end stage of their illness at the time of the interview. Most older participants reportedly saw general practitioners or specialists who spoke their language or had access to interpreters when accessing medical care.

Despite long residency in Australia, English literacy among the participants remained relatively low, and some who spoke English well chose to conduct the interviews in their native language, which suggested a solid attachment to the Chinese language and culture. Consequently many participants relied on their family for support and assistance in navigating the health and aged care systems. Although the participants were open to discussing EOL issues, most had not heard of ACP before their interviews.

The themes identified were:

• The meaning of being alive in the context of serious illness
• Significance of life events
• Role of the individual and family members in decision making
• Social and cultural influences on EOL decision-making
• Attitudes and awareness towards ACP

A compilation of representative quotes for these themes can be found in Table 2.

**Table 2.** Representative Quotations of the Themes – Older Adults and Carers Perspectives.

Theme	Older participants	Carers
The meaning of being alive in the context of serious illness	*“The meaning of being alive is not about living longer… Being alive means accomplishing something, emotionally and mentally being happy. That’s what living is.” (78* *years old, female, Taiwan)*	*“When I cared for my mother, I felt so sorry for her. When under so much suffering, it is cruel to delay death.” (60* *years old, female, Taiwan)*
	“*When you have lost vital functions, your life no longer has meaning. You are just a lump of meat lying there, even though you are still alive.” (76* *years old, male, Mainland China)*	*“Well, the chance may be slim, but there’s still a chance, right?” After the surgery, will he (the patient) recover? Or will he suffer a lot before dying? I think that’s more important to me. I don’t want to see my dad suffer.” (53* *years old, female, Hong Kong)*
Significance of life events	*“It’s very confronting, you sort of never sit down and think about all these things until you see it (death of loved ones) happen in front of your eyes… and sort of like it’s something for me to ponder over my own life.” (65* *years old, male, Malaysia)*	*“If one has never cared for someone who is critically ill and has not observed the pain and suffering of the patient, they may not be able to comprehend why I prayed to the Buddha to take my mother.” (60* *years old, female, Taiwan)*
	*“Just imagine if I fainted suddenly or had cancer, and you took me to the nursing home… Then I’ll end up like my husband, just lying there, waiting to be fed, waiting for the shower after my meals, and then waiting to be fed again. I don’t want this kind of life.” (77* *years old, female, Hong Kong)*	
Role of individual and family members in decision-making	*“It is my own choice. My sons do not have the right to tell me what to do. I don’t need their opinions; decisions are entirely up to me. If I cannot make the ultimate decisions, then it won’t work for me.” (77* *years old, female, Hong Kong)*	*“I think my mom is very aware of what she wants. She has her own mind and she knows what she’s going to do.” (52* *years old, female, Mainland China)*
	*“I made the decision. Well, I discussed it with my wife. I cannot simply act or decide on my own accord. At the same time, they cannot force me to agree with them. It has to be a unanimous decision.” (76* *years old, male, Mainland China)*	*“Actually, he didn’t want to go. But he understood that nursing home is the place to be because my mum and I could not take care of him anymore. It is safer, for his own sake.” (53* *years old, female, Hong Kong)*
	*“so you sort of say okay, you nominated your wife (to be the substitute decision maker), but is she going to do it? Even though this is what you say that you want. And when you're at that stage and when she is at a stage of emotional and whatever, is that possible?”(65* *years old, male, Malaysia)*	*“I was not in the country. My sister asked every single sibling, and we voted. My mother had eight children, all of us were involved, and we voted. We agree unanimously.” (66* *years old, female, Hong Kong)*
Social and cultural influences on EOL decision-making	*“Even if you are well prepared, the younger generation may not choose to obey; therefore, it is fruitless.” (71* *years old, male, China)*	*“I think it is very common in Chinese culture that the parents need to trust their children to make decisions for them. I think it’s not a completely bad thing. You give this hard decision to the children because they are younger, and they are less vulnerable.” (52* *years old, female, China)*
	*“The Chinese customs usually listen to the son. Even if you are the next of kin and have the Power of Attorney, whatever…The son's opinion is more important because they understand more about the doctor's decision. You wouldn't do it by yourself.” (72* *years old, female, Hong Kong)*	
	*“The father’s wishes precede the son’s wishes. So are his mother’s wishes, which should precede the son’s… Rights of the eldest son no longer exist in China.” (76* *years old, male, Mainland China)*	
Attitudes and awareness towards ACP	*“I'm not to that stage yet. Everything is ok at the moment. No dementia yet.” (72* *years old, female, Hong Kong)*	*“But I thought that it's usually that we only need to talk about this when someone is admitted to the hospital or nursing home.” (53* *years old, female, Hong Kong)*
	*“I am in good health now, so even if I am willing to communicate with her (daughter), she may not be able to accept it.” (75* *years old, female, Mainland China)*	*“If I bring this up (directly), they might think, why do you talk about this? Am I dying now? An indirect way to make them think is probably the easiest approach.” (53* *years old, female, Hong Kong)*
	*“And I have everything in place. When I'm still in my good faculty, I write down everything, what I want to do, and what I wish to be done… If I'm taken ill suddenly and can't make any decision, my children know what needs to be done.” (73* *years old, female, Malaysia)*	
	*“I think there should be more promotion. Like in Taiwan, there were talks delivered by health professionals. They explained the importance of the initiative to create public awareness and acceptance.” (78* *years old, female, Taiwan)*	

### The meaning of being alive in the context of serious illness

Participants emphasized self-determination and meaningful engagement with life when talking about EOL. An older participant elaborated, “The meaning of being alive is not about living longer… being alive means accomplishing something, emotionally and mentally being happy. That’s what living is.” (78 years old, female, Taiwan) Both older participants and carers viewed the medical prolongation of life with ongoing suffering and living in total dependence as futile and meaningless. An older participant stated, “When you have lost vital functions, your life no longer has meaning. You are just a lump of meat lying there, even though you are still alive.” (76 years old, male, Mainland China).

Carers, likewise, reported prioritizing avoidance of suffering when considering the risk and benefits of invasive measures at EOL of their loved ones. A carer stated, “When I cared for my mother, I felt so sorry for her. When under so much suffering, it is cruel to delay death.” (60 years old, female, Taiwan).

### Significance of life events

All participants shared life stories involving illnesses of themselves and loved ones, where the participants were directly or indirectly involved in the care delivery and EOL decision-making. Participants posited that these events prompted them to confront their inhibitions towards death and helped them identify what matters most to them at their end of life. An older participant stated, “It’s very confronting. You sort of never sit down and think about all these things until you see it (death of loved ones) happen in front of your eyes… and sort of like it’s something for me to ponder over my own life.” (65 years old, male, Malaysia).

An older participant shared that learning from the hardship she endured while caring for her husband had prompted her to complete an Advance Care Directive (ACD). She stated, “Just imagine if I fainted suddenly or had cancer, and you took me to the nursing home… Then I’ll end up like my husband, just lying there, waiting to be fed, waiting for the shower after my meals, and then waiting to be fed again. I don’t want this kind of life.” (77 years old, female, Hong Kong).

A carer highlighted the significance of life experience in shaping a person’s views and attitudes aptly, “If one has never cared for someone who is critically ill and has not observed the pain and suffering of the patient, they may not be able to comprehend why I prayed to the Buddha to take my mother.” (60 years old, female, Taiwan).

### Role of individual and family members in decision-making

Participants’ views and attitudes on decision-making varied across a broad spectrum. While some participants asserted that dignity and control are the main determinants in decision-making, others maintained that they preferred to make decisions autonomously to avoid burdening their families.

The older participant who completed an ACD before the interview emphasized the importance of control and dignity by stating, “It is my own choice. My sons do not have the right to tell me what to do. I don’t need their opinions; decisions are entirely up to me. If I cannot make the ultimate decisions, then it won’t work for me.” (77 years old, female, Hong Kong).

Some older participants emphasized that one’s EOL decisions should be made in unison with their families, however, the perceived benefits for the family should not override the individual’s wish. Reflecting on recent medical decision-making, an older participant stated, “I made the decision. Well, I discussed it with my wife. I cannot simply act or decide on my own accord. At the same time, they cannot force me to agree with them. It has to be a unanimous decision.” (76 years old, male, Mainland China).

Most carers acknowledged that older people have the right to decision-making since they know their health conditions better than others. Although some carers recalled internal struggles between holding on and letting go, they maintained that the family has the duty and obligation to respect and honour the patient’s wishes.

When prompted to elaborate on the role of the family in EOL decision-making, participants generally asserted that the level of involvement of a family member in the EOL decisions is determined by their role in the family and the intimacy of the relationship. They described a family member who undertakes a primary caring role as having more authority in decision-making than others. Participants also suggested that if the relationship among the family members were intimate and close, they would know the individual's values and preferences and could be entrusted to substitute decision-making.

Most participants posited that a spouse has a duty to support and honour the patient’s wishes. However, some participants speculated on the potential contradiction between the spouses’ and the patient’s desires, which might deter their spouses from executing their EOL wishes.

Some carers observed that the eldest among siblings often had to decide. At the same time, some participants believed the responsibility should not be on one person’s shoulder, and decisions were often reached by voting among the family members. An older participant recalled how an EOL decision was reached among her family, “I was not in the country. My sister asked every single sibling, and we voted. My mother had eight children, all of us were involved, and we voted. We agree unanimously.” (66 years old, female, Hong Kong).

### Social and cultural influences on EOL decision-making

Older participants who emphasized autonomy in decision-making had some characteristics in common. They were female, divorced or widowed and lived alone. Most of them migrated to Australia over 30 years ago, and most of them also had experienced the traumatic death of loved ones or decisional conflicts among family over the death of their loved ones in the past. This cohort of older participants demonstrated a high affinity towards ACP. At the time of the interview, one of these participants already had an ACD. Another had completed a “Do Not Resuscitate” order in Taiwan and said she wanted to complete an ACP in Australia. Two intended to discuss with their children about the nomination of a substitute decision-maker and their EOL preferences. The others had asked for the relevant forms and indicated they would consider ACP in the future.

Most participants acknowledged the Chinese tradition of family duty in decision-making and the prioritized role of a son. However, a few older participants expressed doubts about their wishes being respected and honoured by the younger generation. They also suggested that the privileged rights of the eldest son are no longer in practice.

### Attitudes and awareness towards ACP

Most older participants posited that one should only consider making plans when encountering significant changes in one’s health condition or at risk of losing decision-making capacity.

Some participants reasoned that their family might not be open to the discussion prematurely, and they are likely changing their minds, or their social conditions may change over time. A carer suggested only broaching the subject upon their loved ones’ admission to the hospital or a nursing home. On the other hand, those who displayed high affinity towards ACP distinctively implored it is wise to seize the opportunity while one still has the capacity.

Most participants had not heard about ACP before their interview. When asked about ways to raise awareness of ACP in the Chinese community, many felt it was essential to discuss the subject openly, for example, through community education sessions.

Most participants found the indirect communication strategy of story-telling and scenario-based questions used to conduct the interviews helpful in initiating discussions, prompting reflections and generating insights.

## Discussion

Despite the common belief that death is taboo within the Chinese-speaking communities, we found our participants were open to talking about death and dying and willing to explore the concept of ACP. Consistent with literature suggestions, indirect communication strategies used in the study, i.e., storytelling and scenario-based reflective questions, were effective in facilitating deep reflections and eliciting one’s values, views and perceptions about death and dying.^26,30^ Our participants also indicated that reflection on a variety of significant events, such as events of illnesses or death of loved ones, involvement in EOL decision-making and previous exposure to ACP, have profound influences on their attitudes and perceptions. According to the participants, these experiences helped them overcome the taboo of death, prompted them to ponder their own life, and helped them articulate their values and preferences. The strategy of reflecting on another person’s EOL care experience has also been found to be consistently effective in broaching EOL discussion with Chinese patients in the palliative care setting.^30^

Participants in this study shared similar values and preferences to those from other cultural backgrounds in Australia. They viewed being in a state of total dependence or prolonged suffering as futile and undignified.^31^ This suggests that older Australians from various cultural backgrounds value the quality of life when deciding on EOL matters. Therefore, our findings emphasize the importance of framing EOL discussions around what one values most in life rather than focusing solely on future health trajectories and treatment options.^16,31,32^

This study found that participants’ attitudes and perceptions towards ACP were profoundly influenced by their past experiences of involvement in EOL care and decision-making.^1,13,33,34^ In this study, the participants who had experienced decisional conflicts and those who had witnessed the traumatic or sudden death of loved ones were found to display self-determination and high affinity towards ACP. This observation supports the current literature, which posits that the experience of decisional conflicts among family members, the associated emotional burden and having witnessed the suffering induced by the decisions are associated with higher affinity towards ACP.^35,36^

While exercising autonomy and control, most older participants also valued their family's involvement and consensual agreements on their decisions. The findings are consistent with the existing literature on the value of collective or familial decision-making.^20,22,37^ Our findings are demonstrably consistent with the findings of Yap et al^10^ who suggested that individual and familial decision-making are not mutually exclusive.

When reflecting on the role and responsibility of family members in EOL decision-making, many participants looked to their spouses or the eldest children as their main collaborators in decision-making. Contrary to the Chinese tradition of family duty in decision-making and the prioritized role of a son, some participants posited that the privilege and responsibility of making significant decisions no longer rests on the shoulder of the eldest son. Instead they suggested that family members who undertook the primary carer role should have the authority to be the substitute decision maker, and families may consider collective decision-making through voting. Our findings suggest that attitudes and perceptions of Chinese-speaking participants were broadly consistent with the structures embedded in the ‘hierarchy of persons responsible’ in NSW, which depicted the primary non-paid carer as having precedence over a close relative (e.g., son) in substitute decision making.^6^ These findings indicate the importance of including the family members in the ACP discussions and to listen to their thoughts, fears and perception on the patient’s decisions. Current literature suggested that when a spouse or family member is being listened to, they are more likely to honour the patient’s preferences.^38,39^ Therefore, consistent with the literature, we recommend to allow for both parties to have their perspectives heard and to consider how the decisions about one may affect the other as a part of the ACP discussion.^38,39^ Our findings also emphasized the importance of considering the family dynamics and addressing the other family members' concerns and conflicting interests in future health-related discussions and planning.^20,22,37,40^

Many studies have observed the shift of views and attitudes on EOL decisions and ACP of migrants in Western countries towards the Western mindset as they integrated with the host nation’s dominant culture over time.^36,41,42^ We found that although the “self-determination” mindset was observable, and participants reportedly had access to health services in their preferred languages, their awareness and uptake of ACP remained low due to the lack of promotion of ACP within the community. Yap et al. posited that the cultural taboo of discussing death and the preference for familial decision-making might have deterred community leaders and providers from broaching ACP with their community members. Community leaders and aged care providers serving the Chinese-speaking community play a crucial role as custodians in maintaining cultural identity and connectedness within the cultural network.^43,44^ Therefore, they are integral in championing EOL issues and ACP awareness. The study participants have indicated a need for public awareness campaigns and publicly accessible forums and seminars on ACP to help raise community awareness.

Although the participants in our study who were older women, divorced or widowed and lived alone demonstrated a high affinity towards ACP, the small sample size (six male participants) and the use of convenience sampling preclude making generalising statements about this observation. However the significance of gender identity in decision-making at older age deserves further investigation as previous studies found a similarly higher prevalence of ACP among older widowed females in Australia and the United States.^14,45^ Our findings suggest that health and aged care providers should recognize the needs of older adults who live alone in planning and deciding their future healthcare needs autonomously.

### Strength and limitations

Using Mandarin and Cantonese in the interviews, we presented the perspectives of people who may be less familiar with the Australian healthcare system or have different perspectives and needs than people who might be fluent in English. Although the sample provided rich and in-depth insights into the research topics, it was restricted to Sydney metropolitan area, where Mandarin and Cantonese are spoken more prominently. There was also an overrepresentation of Cantonese-speaking participants due to snowball referral. Therefore, the outcomes may not transfer to Chinese-speaking Australians who speak a different dialect or reside in other settings such as residential aged care homes, hospitals or rural and regional settings.

### Implications for practice and future research

This study suggests that healthcare professionals (HCP) and service providers working with Chinese-speaking older adults should identify opportunities for initiating ACP discussions with their clients.

We recommend the use of depersonalised communication strategy to initiatie ACP discussions and the discussion should centred around what older adults value most in life and their past experiences concerning illnesses or EOL decision-making. The discussion should also include family members in ACP discussions where possible.

Finally, we want to highlight the importance of translational research to support the community in putting the above implications and recommendations into practice.

## Appendix

### Appendix 1 Interview guide

**Table:**

Interview questions
**Part one:**
I would like to know more about your background, and the events that have been important for you in your life.
Prompts:
• Migration journey
• Your role(s) in the family
• Health conditions and care needs – personal and family
**Mr Zheng’s story and prompts**
Mr Zheng is 79 years old. Despite being seriously unwell and mostly wheelchair bound for nearly a year, he has been managing to stay at home with the help of his wife (Mrs Zheng) and son (David), and a visiting home care service. He has said on a number of occasions that he wants to stay at home and doesn’t want to be in an aged care home. He also once said that if Mrs Zheng needed to make decisions for him, he would prefer to have a natural death, rather than be kept alive by machines in the hospital. But Mrs Zheng said they should not talk about things like that.
One day Mr Zheng becomes suddenly more unwell. He is semi-conscious and appears to have pain in his stomach area. Mrs Zheng called David, who called the ambulance. In the hospital the doctors examined Mr Zheng and said that urgent surgery would be needed to save his life. However, given Mr Zheng’s frailty, the chances of him recovering from the surgery and being able to go home were very low.
Does this remind you of any stories you have been aware of in your community or family?
Imagine you were in Mr Zheng’s situation. What would be most important to you?
David came immediately to the hospital, but Mrs Zheng did not follow along. In the absence of Mrs Zheng, David insisted that he should be the one making the decision as he is the eldest son. He also said that it is his duty to make sure his father receives the best treatment. David said that the doctors should do everything possible to save Mr Zheng’s life. When asked about Mrs Zheng, David said his mother did not want to come to the hospital because she believes that she will catch all sorts of diseases. She also feels very disempowered because she doesn’t speak English and often does not understand the forms she has been asked to sign.
Does this remind you of any stories you have been aware of in your community or family?
Imagine if you were in David or Mrs Zheng’s situation. What would be most important?
**Part two:**
In Australia, there are laws that require the doctors to ask permission before starting medical treatments or procedures. Normally they would ask the patient who they intend to give the treatment to. In this research we are trying to understand what normally happens in the Chinese-speaking community when these serious medical decisions are required. If you know of any stories that relate to this, can you tell me? From your experience, can you tell me about:
Is it usually the patient who makes decisions about their care/treatment? Or other people
How are these decisions usually made? What do people take into account when making these sorts of decisions?
What usually happens if the patient can’t communicate or make their own decisions?
What usually happens if some people don’t speak English?
In Australia, it is encouraged for people to talk about what they would want for their future healthcare, in case there was a time when they could not speak for themselves or make decisions. This is called advance care planning. People over the age of eighteen can also complete a legal document, to nominate who they would want to make decisions for them (Enduring Guardianship) or to state what treatments they would or would not want (Advance Care Directive).
Have you heard about advance care planning before? Do you have any experience with this?
What are your views on advance care planning?
Thinking about the Chinese speaking community in Australia, what do you think about their views on advance care planning?
What could be done to make advance care planning work better for the Chinese speaking community?
